# Supplementary material for: Phenotypic diversity of Methylobacterium associated with rice landraces in North-East India
Source: PLoS One. 2020 Feb 24;15(2):e0228550. doi: 10.1371/journal.pone.0228550 (PMC7039438; doi:10.1371/journal.pone.0228550)
Supplement: S1 Table — Sampling states are abbreviated as AR (Arunachal Pradesh) and MN (Manipur). (DOCX) [file pone.0228550.s002.docx]

**S1 Table**: List of distinct *Methylobacterium* isolates sampled in September – October 2016. Sampling states are abbreviated as AR (Arunachal Pradesh) and MN (Manipur).

| **SI no.** | **State** | **Landrace** | **Isolate** | **Closest identified species** |
| --- | --- | --- | --- | --- |
| 1 | AR | Amkil (AMK) | AMKL1 | *Methylobacterium aminovorans* |
| 2 | AR | Amkil (AMK) | AMKS1 | *Methylobacterium radiotolerans* |
| 3 | AR | Amham (AMH) | AMHL1 | *Methylobacterium aquaticum* |
| 4 | AR | Amham (AMH) | AMHS1 | *Methylobacterium radiotolerans* |
| 5 | AR | Ammo (AMM) | AMML1 | *Methylobacterium aerolata* |
| 6 | AR | Ammo (AMM) | AMMS5 | *Methylobacterium aerolata* |
| 7 | AR | Deku (DK) | DKS6 | *Methylobacterium salsuginis* |
| 8 | AR | Deku (DK) | DKL1 | *Methylobacterium p.53* |
| 9 | AR | Deku (DK) | DKL2 | *Methylobacterium rhodinum* |
| 10 | AR | Essum (ESS) | ESSS2 | *Methylobacterium lusitanum* |
| 11 | AR | Essum (ESS) | ESSL2 | *Methylobacterium rhodinum* |
| 12 | AR | Essum (ESS) | ESSS4 | *Methylobacterium salsuginis* |
| 13 | AR | Gegong (GEG) | GEGL2 | *Methylobacterium radiotolerans* |
| 14 | AR | Gegong (GEG) | GEGS2 | *Methylobacterium salsuginis* |
| 15 | AR | Gegong (GEG) | GEGS6 | *Methylobacterium rhodinum* |
| 16 | AR | Gegong (GEG) | GEGS4 | *Methylobacterium extorquens* |
| 17 | AR | Gezang (GEZ) | GEZL2 | *Methylobacterium radiotolerans* |
| 18 | AR | Gezang (GEZ) | GEZS2 | *Methylobacterium radiotolerans* |
| 19 | AR | Dalmang (DAL) | DALS4 | *Methylobacterium radiotolerans* |
| 20 | AR | Dalmang (DAL) | DALL2 | *Methylobacterium salsuginis* |
| 21 | AR | Dalmang (DAL) | DALS6 | *Methylobacterium zatmani* |
| 22 | AR | Nelii black (NELB) | NELBL2 | *Methylobacterium aquaticum* |
| 23 | AR | Nelii black (NELB) | NELBS1 | *Methylobacterium aerolata* |
| 24 | AR | Nelii black (NELB) | NELBS5 | *Methylobacterium komagatae* |
| 25 | AR | Itanagar rice (IT) | ITS5 | *Methylobacterium radiotolerans* |
| 26 | AR | Itanagar rice (IT) | ITS2 | *Methylobacterium salsuginis* |
| 27 | AR | Pallu nelii (PLN) | PLNL1 | *Methylobacterium fujisawaense* |
| 28 | AR | Pallu nelii (PLN) | PLNL2 | *Methylobacterium phyllosphaerae* |
| 29 | AR | Pallu nelii (PLN) | PLNS3 | *Methylobacterium aquaticum* |
| 30 | AR | Pallu nelii (PLN) | PLNS5 | *Methylobacterium aerolata* |
| 31 | AR | Pyapiimepha (PMPHA) | PMPHAS6 | *Methylobacterium fujisawaense* |
| 32 | AR | Pyapiimepha (PMPHA) | PMPHAS1 | *Methylobacterium radiotolerans* |
| 33 | AR | Pyapii (PYA) | PYAL1 | *Methylobacterium komagatae* |
| 34 | AR | Pyapii (PYA) | PYAL2 | *Methylobacterium suomiense* |
| 35 | AR | Pyapii (PYA) | PYAS1 | *Methylobacterium komagatae* |
| 36 | AR | Pangi amm (PAN) | PANS4 | *Methylobacterium radiotolerans* |
| 37 | AR | Pangi amm (PAN) | PANL1 | *Methylobacterium salsuginis* |
| 38 | AR | Rasing (RAS) | RASL2 | *Methylobacterium aerolata* |
| 39 | AR | Rasing (RAS) | RASL1 | *Methylobacterium komagatae* |
| 40 | AR | Taker (TAK) | TAKS2 | *Methylobacterium populi* |
| 41 | AR | Taker (TAK) | TAKL2 | *Methylobacterium populi* |
| 42 | AR | Tuyim (TUY) | TUYS3 | *Methylobacterium salsuginis* |
| 43 | AR | Tuyim (TUY) | TUYS5 | *Methylobacterium suomiense* |
| 44 | AR | Yagrun (YG) | YGL1 | *Methylobacterium radiotolerans* |
| 45 | AR | Yagrun (YG) | YGS2 | *Methylobacterium radiotolerans* |
| 46 | AR | Yakil (YAK) | YAKL1 | *Methylobacterium salsuginis* |
| 47 | AR | Yakil (YAK) | YAKS1 | *Methylobacterium salsuginis* |
| 48 | AR | Tusing (TUS) | TUSS6 | *Methylobacterium zatmanii* |
| 49 | AR | Tusing (TUS) | TUSS2 | *Methylobacterium komagatae* |
| 50 | AR | Tusing (TUS) | TUSL2 | *Methylobacterium rhodinum* |
| 51 | MN | Abung phou (AP) | APS4 | *Methylobacterium suomiense* |
| 52 | MN | Abung phou (AP) | APS5 | *Methylobaterium aqaumaris* |
| 53 | MN | Abung phou (AP) | APL2 | *Methylobacterium radiotolerans* |
| 54 | MN | Chakhao 1 (CK1) | CK1L1 | *Methylobacterium aquaticum* |
| 55 | MN | Chakhao 1 (CK1) | CK1S3 | *Methylobacterium suomiense* |
| 56 | MN | Chakhao 2 (CK2) | CK2S2 | *Methylobacterium komagatae* |
| 57 | MN | Chakhao 2 (CK2) | CK2L1 | *Methylobacterium suomiense* |
| 58 | MN | Chakhao 3 (CK3) | CK3S3 | *Methylobacterium suomiense* |
| 59 | MN | Chakhao 3 (CK3) | CK3S4 | *Methylobacterium suomiense* |
| 60 | MN | Chakhao pireiton (CKP) | CKPL2 | *Methylobacterium salsuginis* |
| 61 | MN | Chakhao pireiton (CKP) | CKPS1 | *Methylobacterium aquaticum* |
| 62 | MN | Kakcheng phou (KAK) | KAKL1 | *Methylobacterium populi* |
| 63 | MN | Kakcheng phou (KAK) | KAKS3 | *Methylobacterium phyllosphaerae* |
| 64 | MN | Kakcheng phou (KAK) | KAKS1 | *Methylobacterium radiotolerans* |
| 65 | MN | Kumbi phou (KUM) | KUMS6 | *Methylobacterium komagatae* |
| 66 | MN | Kumbi phou (KUM) | KUML2 | *Methylobacterium radiotolerans* |
| 67 | MN | Kumbi phou (KUM) | KUMS5 | *Methylobacterium radiotolerans* |
| 68 | MN | Langphou (LAN) | LANL1 | *Methylobacterium salsuginis* |
| 69 | MN | Langphou (LAN) | LANS1 | *Methylobacterium salsuginis* |
| 70 | MN | Khokngangbi (KHOK) | KHOKL1 | *Methylobacterium suomiense* |
| 71 | MN | Khokngangbi (KHOK) | KHOKS1 | *Methylobacterium komagatae* |
| 72 | MN | Moirangphou (MAN) | MANS3 | *Methylobacterium oryzae* |
| 73 | MN | Moirangphou (MAN) | MANS6 | *Methylobacterium radiotolerans* |
| 74 | MN | Moirangphou (MAN) | MANL2 | *Methylobacterium radiotolerans* |
| 75 | MN | Moirangphou (MAN) | MANL1 | *Methylobacterium salsuginis* |
| 76 | MN | Phouren-mubi (PM) | PML2 | *Methylobacterium radiotolerans* |
| 77 | MN | Phouren-mubi (PM) | PMS2 | *Methylobacterium komagatae* |
| 78 | MN | Phouren-mubi (PM) | PMS1 | *Methylobacterium radiotolerans* |
| 79 | MN | Phou-ngang (PN) | PNS3 | *Methylobacterium persicinum* |
| 80 | MN | Phou-ngang (PN) | PNS6 | *Methylobacterium aquaticum* |
| 81 | MN | Phou-ngang (PN) | PNL1 | *Methylobacterium salsuginis* |
| 82 | MN | Taothabi (TAO) | TAOS1 | *Methylobacterium salsuginis* |
| 83 | MN | Taothabi (TAO) | TAOS5 | *Methylobacterium aquaticum* |
| 84 | MN | Taothabi (TAO) | TAOL1 | *Methylobacterium radiotolerans* |
| 85 | MN | Tolen phou (TP) | TPL1 | *Methylobacterium salsuginis* |
| 86 | MN | Tolen phou (TP) | TPS5 | *Methylobacterium aquaticum* |
| 87 | MN | Tolen phou (TP) | TPS2 | *Methylobacterium lusitanum* |
| 88 | MN | Akhan phou (AKP) | AKANL1 | *Methylobacterium aquaticum* |
| 89 | MN | Akhan phou (AKP) | AKANS5 | *Methylobacterium salsuginis* |
| 90 | MN | Laiphou (LAP) | LAPL1 | *Methylobacterium salsuginis* |
| 91 | MN | Laiphou (LAP) | LAPS1 | *Methylobacterium salsuginis* |
