# Supplementary material for: Phenotypic diversity of Methylobacterium associated with rice landraces in North-East India
Source: PLoS One. 2020 Feb 24;15(2):e0228550. doi: 10.1371/journal.pone.0228550 (PMC7039438; doi:10.1371/journal.pone.0228550)
Supplement: S2 Table — All abbreviations used for landraces are given in brackets after the landrace name. (DOCX) [file pone.0228550.s003.docx]

**S2 Table**: List of distinct *Methylobacterium* isolates sampled from seven focal landraces of Manipur in September 2017. All the abbreviations used for landrace is given in brackets after the landrace name.

| **Sl.no.** | **Landrace** | **Isolates** | **Closest identified species** |
| --- | --- | --- | --- |
| 1 | Chakhao (CKP) | CKPL1 | *Methylobacterium salsuginis* |
| 2 | Chakhao (CKP) | CKPL2 | *Methylobacterium aerolatum* |
| 3 | Chakhao (CKP) | CKPL3 | *Methylobacterium aerolatum* |
| 4 | Chakhao (CKP) | CKPS4 | *Methylobacterium salsuginis* |
| 5 | Chakhao (CKP) | CKPL5 | *Methylobacterium gossipicola* |
| 6 | Phouren-mubi (PM) | PML1 | *Methylobacterium suomiense* |
| 7 | Moirangphou (MAN) | MANL1 | *Methylobacterium suomiense* |
| 8 | Moirangphou (MAN) | MANL2 | *Methylobacterium sp.P53* |
| 9 | Moirangphou (MAN) | MANL3 | *Methylobacterium phyllospherae* |
| 10 | Moirangphou (MAN) | MANL4 | *Methylobacterium komagate* |
| 11 | Kumbi-phou (KUM) | KUML1 | *Methylobacterium komagatae* |
| 12 | Kumbi-phou (KUM) | KUMS2 | *Methylobacterium komagatae* |
| 13 | Kumbi-phou (KUM) | KUML3 | *Methylobacterium sp.P53* |
| 14 | Kumbi-phou (KUM) | KUMS4 | *Methylobacterium suomiense* |
| 15 | Kumbi-phou (KUM) | KUMS5 | *Methylobacterium salsuginis* |
| 16 | Kumbi-phou (KUM) | KUMS6 | *Methylobacterium komagatae* |
| 17 | Phou-ngang (PN) | PNS1 | *Methylobacterium indicum* |
| 18 | Langphou (LAN) | LANS1 | *Methylobacterium aerolatum* |
| 19 | Langphou (LAN) | LANL2 | *Methylobacterium komagate* |
| 20 | Langphou (LAN) | LANL3 | *Methylobacterium radiotolerans* |
| 21 | Abung phou (AP) | APL1 | *Methylobacterium sp. 9HR-3* |
| 22 | Abung phou (AP) | APL2 | *Methylobacterium komagatae* |
| 23 | Abung phou (AP) | APS3 | *Methylobacterium salsuginis* |
| 24 | Abung phou (AP) | APL4 | *Methylobacterium sp. P53* |
