# Supplementary material for: Phenotypic diversity of Methylobacterium associated with rice landraces in North-East India
Source: PLoS One. 2020 Feb 24;15(2):e0228550. doi: 10.1371/journal.pone.0228550 (PMC7039438; doi:10.1371/journal.pone.0228550)
Supplement: S3 Table — (DOCX) [file pone.0228550.s004.docx]

**S3 Table:** List of *Methylobacterium* isolates sampled from landraces, seeds, soil, grasses, and commercial rice cultivated in the Chingarel field, Manipur.

| **Sl.no.** | **Source** | **Sample** | **Isolate** | **Closest identified Species** |
| --- | --- | --- | --- | --- |
| 1 | Soil | Soil | C1 | *Methylobacterium radiotolerans* |
| 2 | Soil | Soil | C3 | *Methylobacterium radiotolerans* |
| 3 | Soil | Soil | C5 | *Methylobacterium radiotolerans* |
| 4 | Landrace | Abung phou | APS4 | *Methylobacterium suomiense* |
| 5 | Landrace | Abung phou | APS5 | *Methylobacterium aquamaris* |
| 6 | Landrace | Abung phou | APL2 | *Methylobacterium radiotolerans* |
| 7 | Landrace | Chakhao Poireiton | CKPL2 | *Methylobacterium salsuginis* |
| 8 | Landrace | Chakhao Poireiton | CKPS1 | *Methylobacterium aquaticum* |
| 9 | Landrace | Phouren mubi | PML2 | *Methylobacterium radiotolerans* |
| 10 | Landrace | Phouren mubi | PMS2 | *Methylobacterium komagatae* |
| 11 | Landrace | Phouren mubi | PMS1 | *Methylobacterium radiotolerans* |
| 12 | Landrace | Moirang-phou | MANS3 | *Methylobacterium oryzae* |
| 13 | Landrace | Moirang-phou | MANS6 | *Methylobacterium radiotolerans* |
| 14 | Landrace | Moirang-phou | MANL2 | *Methylobacterium radiotolerans* |
| 15 | Landrace | Moirang-phou | MANL1 | *Methylobacterium salsuginis* |
| 16 | Seed | Chakhao Poireiton | CK1 | *Methylobacterium radiotolerans* |
| 17 | Seed | Chakhao Poireiton | CK2 | *Methylobacterium radiotolerans* |
| 18 | Seed | Phouren mubi | PM1 | *Methylobacterium radiotolerans* |
| 19 | Seed | Moirang-phou | MP2 | *Methylobacterium radiotolerans* |
| 20 | Seed | Moirang-phou | MP4 | *Methylobacterium radiotolerans* |
| 21 | GR | Grass | GL3.1 | *Methylobacterium rhodinum* |
| 22 | GR | Grass | GL3.2 | *Methylobacterium salsuginis* |
| 23 | GR | Grass | GL3.4 | *Methylobacterium radiotolerans* |
| 24 | GR | Grass | GL2.1 | *Methylobacterium radiotolerans* |
| 25 | HYV-701 | Commercial rice | HYV-2 | *Methylobacterium salsuginis* |
| 26 | HYV-701 | Commercial rice | HYV-4 | *Methylobacterium salsuginis* |
| 27 | HYV-701 | Commercial rice | HYV-1 | *Methylobacterium radiotolerans* |
| 28 | HYV-701 | Commercial rice | HYV-3 | *Methylobacterium salsuginis* |
