# Supplementary material for: Phenotypic diversity of Methylobacterium associated with rice landraces in North-East India
Source: PLoS One. 2020 Feb 24;15(2):e0228550. doi: 10.1371/journal.pone.0228550 (PMC7039438; doi:10.1371/journal.pone.0228550)
Supplement: S4 Table — (DOCX) [file pone.0228550.s005.docx]

**S4 Table:** List of distinct *Methylobacterium* isolates sampled from the seeds of landraces from Arunachal Pradesh (AR) and Manipur (MN).

| **Sl.no.** | **State** | **Landrace** | **Isolate** | **Closest identified species** |
| --- | --- | --- | --- | --- |
| 1 | AR | Amham (AMH) | AMH3 | *Methylobacterium salsuginis* |
| 2 | AR | Amkil (AMK) | AMK2 | *Methylobacterium fujisawaense* |
| 3 | AR | Amkil (AMK) | AMK3 | *Methylobacterium fujisawaense* |
| 4 | AR | Gegong (GEG) | GEG1 | *Methylobacterium aminovorans* |
| 5 | AR | Gegong (GEG) | GEG2 | *Methylobacterium aquaticum* |
| 6 | AR | Gezang (GEZ) | GEZ2 | *Methylobacterium radiotolerans* |
| 7 | AR | Yagrun (YG) | YG1 | *Methylobacterium radiotolerans* |
| 8 | AR | Yagrun (YG) | YG2 | *Methylobacterium radiotolerans* |
| 9 | MN | Chakhao poireiton | CKP | *Methylobacterium radiotolerans* |
| 10 | MN | Kumbi-phou (KUM) | KUM1 | *Methylobacterium radiotolerans* |
| 11 | MN | Moirang-phou (MAN) | MP2 | *Methylobacterium radiotolerans* |
| 12 | MN | Moirang-phou (MAN) | MP4 | *Methylobacterium radiotolerans* |
| 13 | MN | Phouren-mubi (PM) | PM1 | *Methylobacterium radiotolerans* |
| 14 | MN | Phou-ngang (PN) | PN | *Methylobacterium radiotolerans* |
| 15 | MN | Phou-ngang (PN) | PN1 | *Methylobacterium radiotolerans* |
