# Supplementary material for: Phenotypic diversity of Methylobacterium associated with rice landraces in North-East India
Source: PLoS One. 2020 Feb 24;15(2):e0228550. doi: 10.1371/journal.pone.0228550 (PMC7039438; doi:10.1371/journal.pone.0228550)
Supplement: S5 Table — (DOCX) [file pone.0228550.s006.docx]

**S5 Table:** Repeatability of carbon utilization profile of *Methylobacterium* isolates, tested for a random subset of isolates across two experimental blocks.

| Total isolates | Number of mismatches | | | |
| --- | --- | --- | --- | --- |
|  | Glucose | Fructose | Xylose | Sucrose |
| 30 | 1 | 5 | 2 | 0 |
