# Supplementary figures and images for: Phenotypic diversity of Methylobacterium associated with rice landraces in North-East India
Source: PLoS One. 2020 Feb 24;15(2):e0228550. doi: 10.1371/journal.pone.0228550 (PMC7039438; doi:10.1371/journal.pone.0228550)

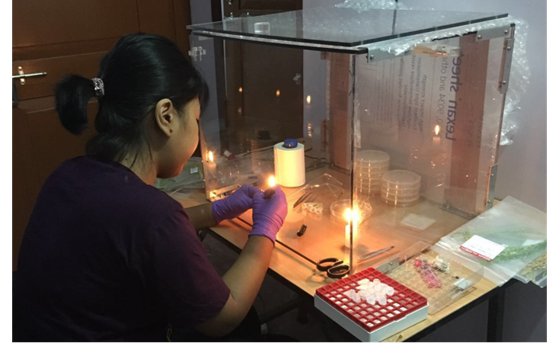

Supplement: S1 Fig — (TIF) [file pone.0228550.s007.tif]

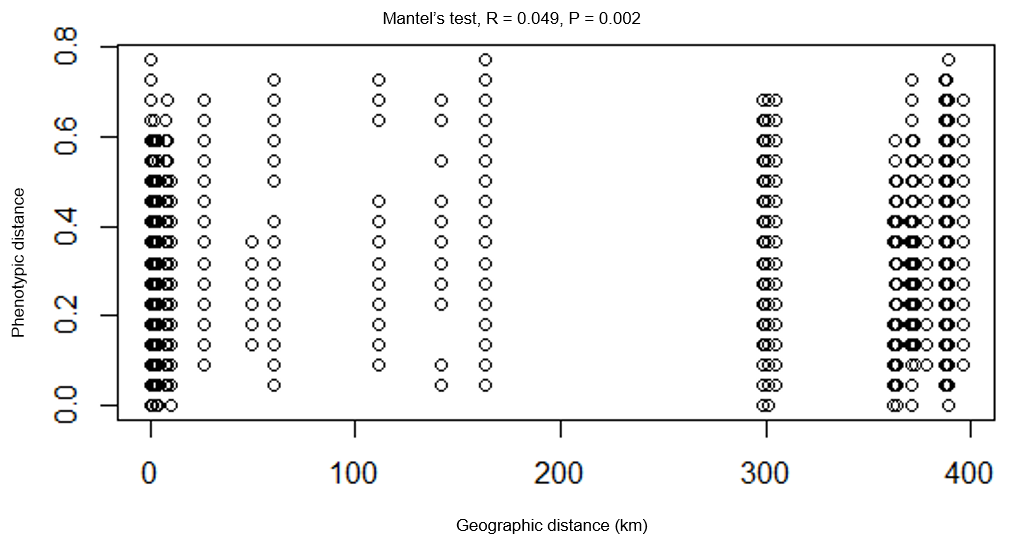

Supplement: S2 Fig — Scatter plot showing pairwise phenotypic distance vs. geographic distance for 91 distinct Methylobacterium isolates. (TIF) [file pone.0228550.s008.tif]

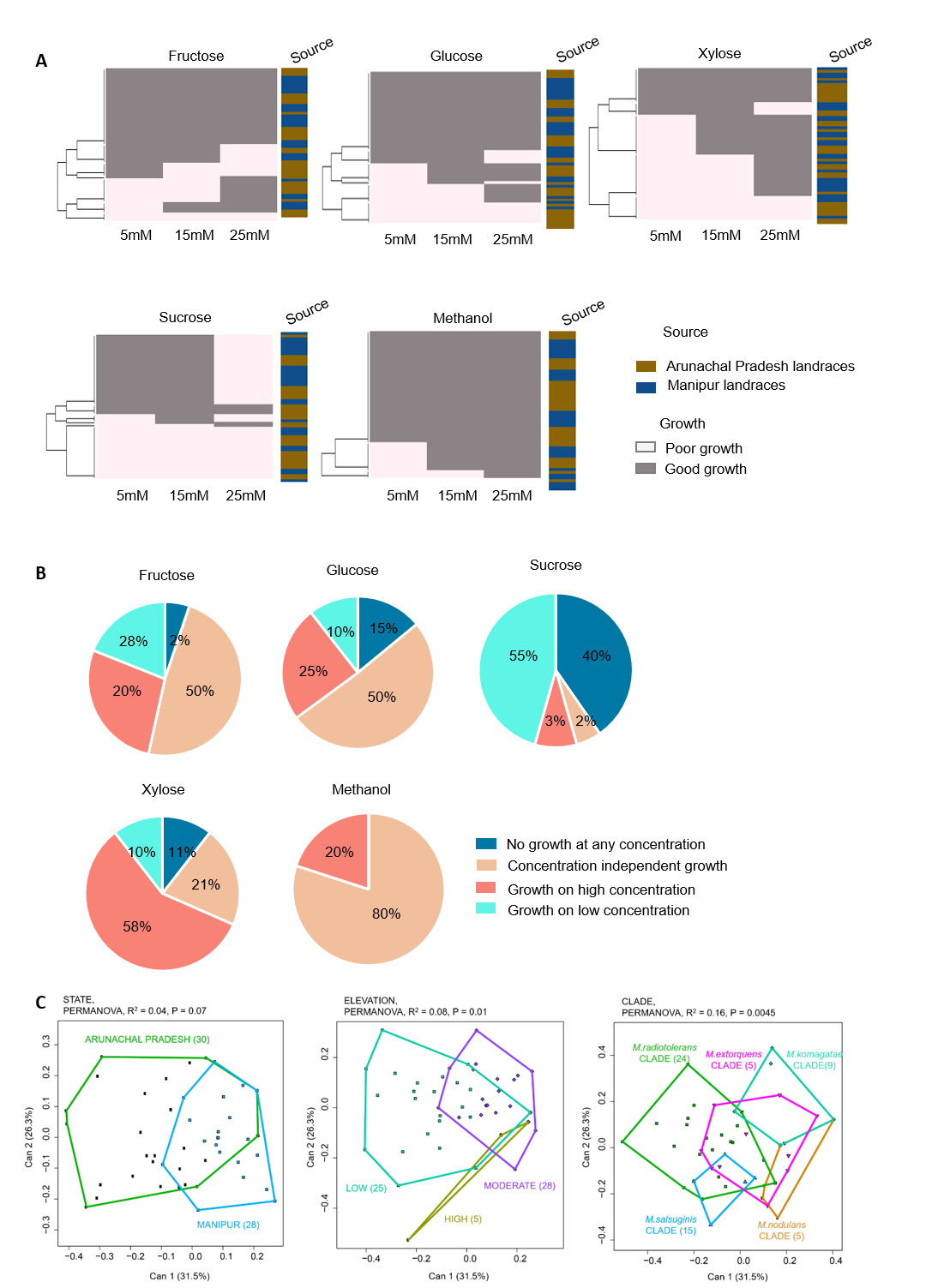

Supplement: S3 Fig — (A) A summary of the growth of Methylobacterium (58 isolates) on different concentrations of fructose, glucose, xylose, sucrose, and methanol. (B) Fraction of Methylobacterium showing different patterns of growth on each carbon source (see key for details). (C) Canonical variance analysis biplots of carbon use profiles, showing the clustering of Methylobacterium isolates by sampling state, elevation, and Methylobacterium clade. (TIF) [file pone.0228550.s009.tif]

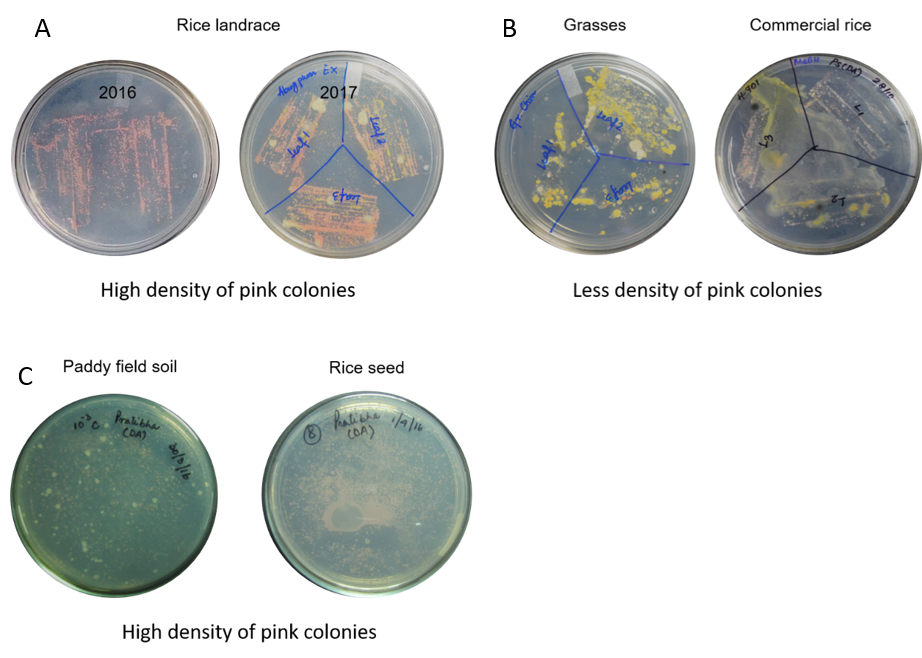

Supplement: S4 Fig — Samples were imprinted or dilution-plated on Hypho minimal agar plates with 120mM methanol as the sole carbon source. Pink colonies are typical of Methylobacterium sp. (TIF) [file pone.0228550.s010.tif]

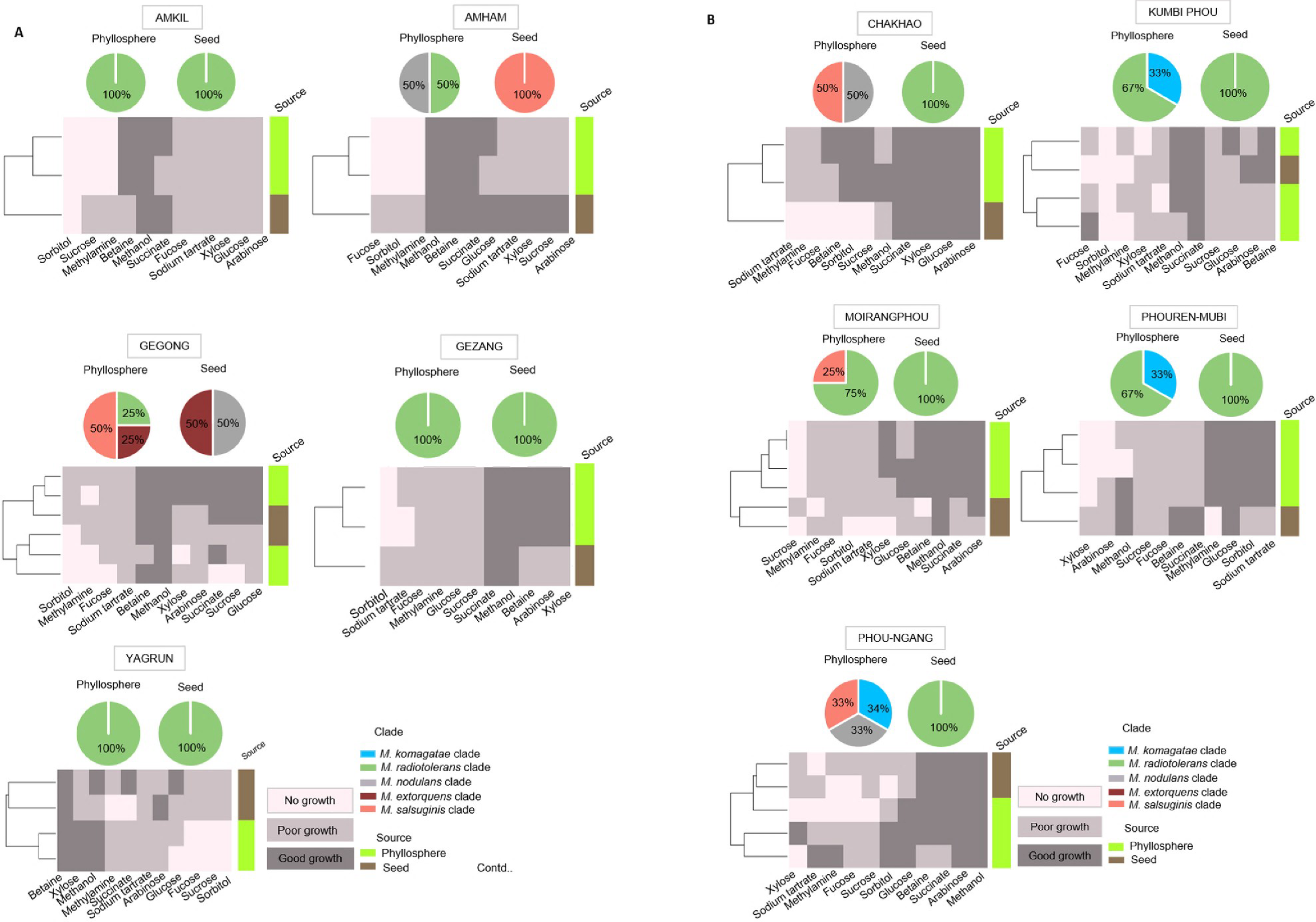

Supplement: S5 Fig — Each panel shows a pairwise comparison of the Methylobacterium community associated with phyllosphere vs. seed for a given each landraces, both in terms of community composition (piecharts) and carbon use phenotype (heatmap). (A) Landraces sampled in Arunachal Pradesh; (B) Landraces sampled in Manipur. (TIF) [file pone.0228550.s011.tif]

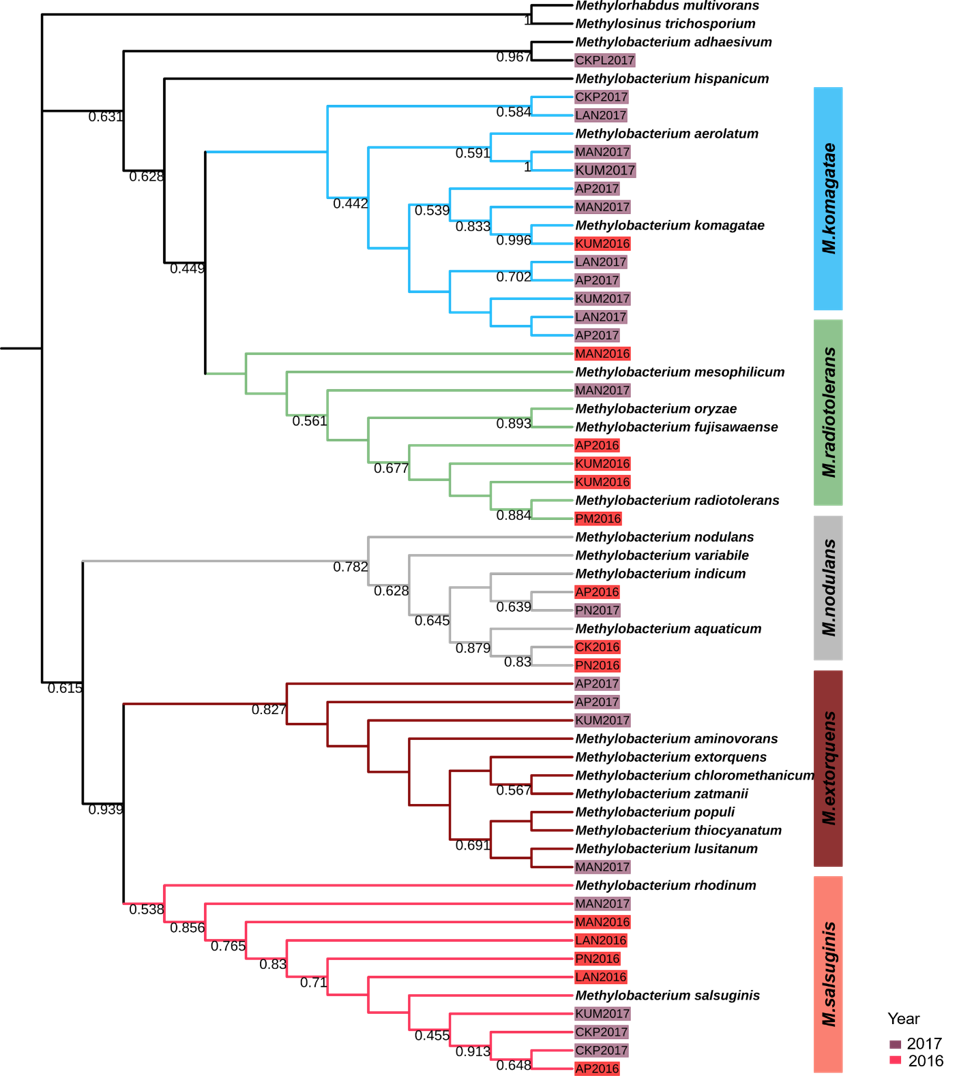

Supplement: S6 Fig — Neighbor-joining tree constructed from the 16S rRNA gene sequences of distinct Methylobacterium isolates from rice landraces (colored by sampling year), and closely related reference strains (in bold). Bootstrap values ≥ 50% are indicated. Clades are indicated on the right (see Fig 2). (TIF) [file pone.0228550.s012.tif]

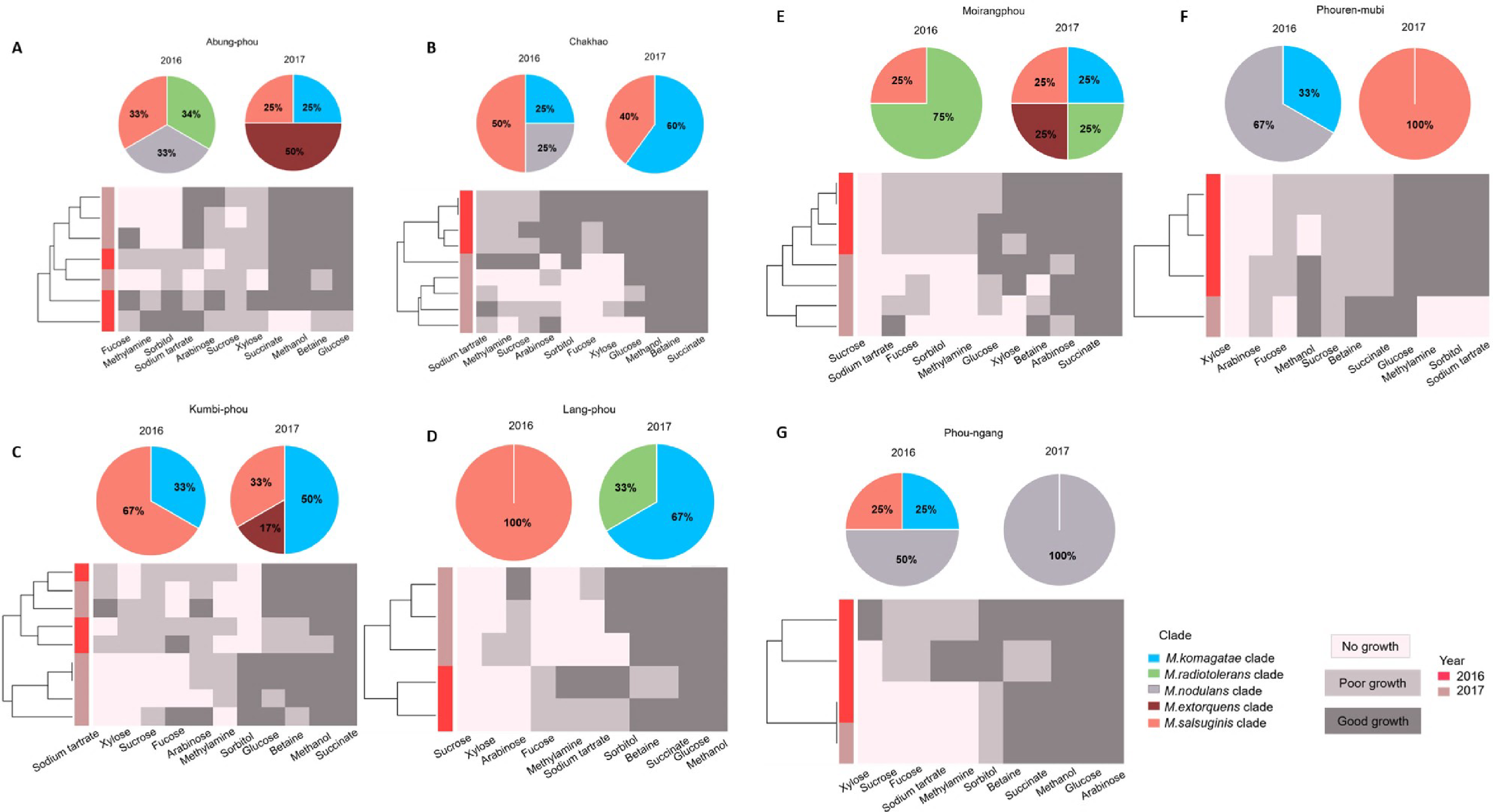

Supplement: S7 Fig — Each panel shows a pairwise comparison between bacterial isolates from a given landrace sampled in 2016 vs. 2017 from Manipur, both in terms of community composition (piecharts) and carbon use profile (heatmap). (TIF) [file pone.0228550.s013.tif]

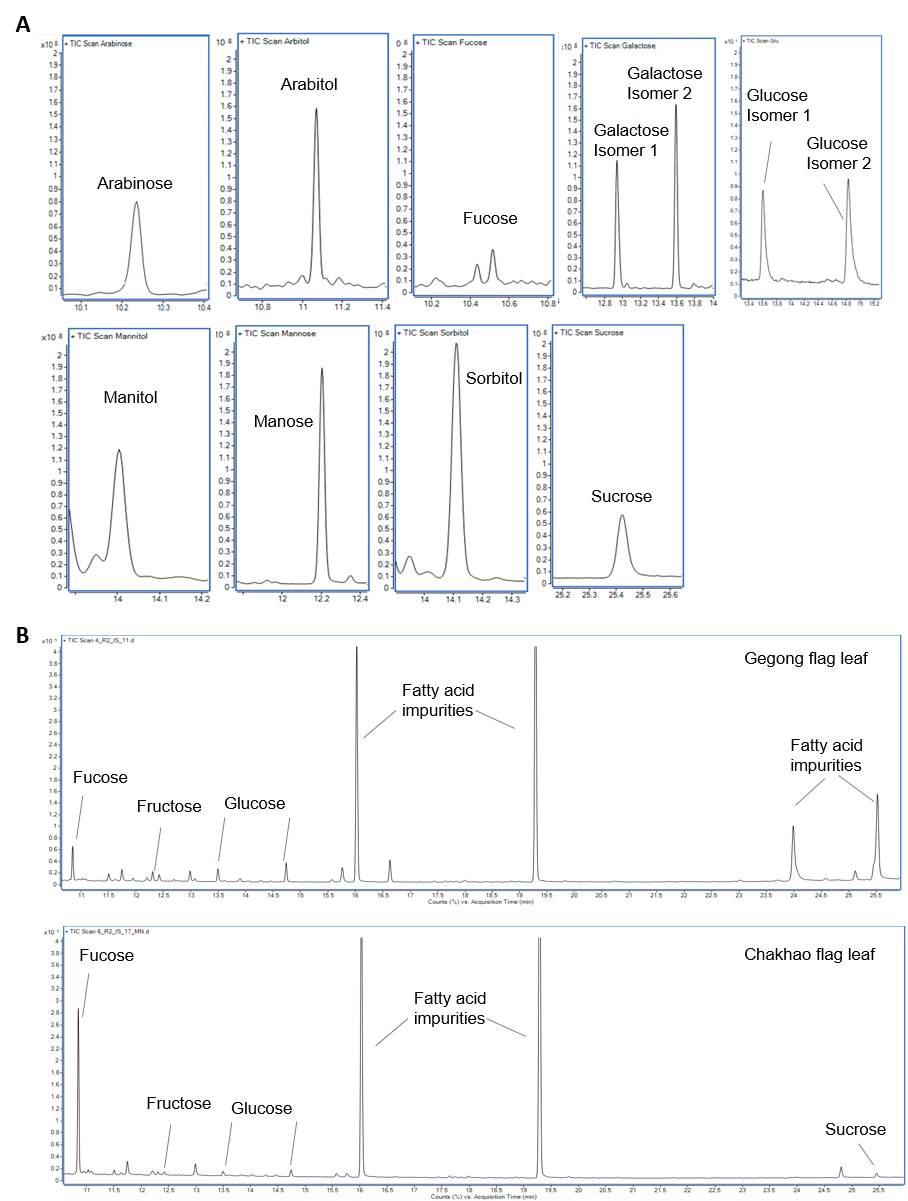

Supplement: S8 Fig — (A) GCMS chromatograms of standard sugars. (B) Representative chromatograms of flag leaf exudates of two landraces: Gegong (Arunachal Pradesh) and Chakhao (Manipur). (TIF) [file pone.0228550.s014.tif]
