# Supplementary material for: Phenotypic diversity of Methylobacterium associated with rice landraces in North-East India
Source: PLoS One. 2020 Feb 24;15(2):e0228550. doi: 10.1371/journal.pone.0228550 (PMC7039438; doi:10.1371/journal.pone.0228550)
Supplement: Attachment — Submitted filename: Response to reviewers_20191203.docx [file pone.0228550.s015.docx]

**Response to reviewers**

**Reviewer #1: The author and the team did very good work in the field, lab, and compilation. However, few things need to be improved.**

**1. Northeast India is a vast area comprising of many states. Why only two states (Manipur and Arunachal Pradesh) were chosen for sample collection? The states of Assam and Tripura are major rice-producing states of the region and more *Methylobacterium* strains could have been isolated for better resolution.**

For various logistical reasons, we restricted our sampling to two states where farmers cultivate a large number of traditional rice varieties (rather than commercial high-yielding varieties); where sample transport and laboratory access was feasible; and where we already had local contacts who could facilitate field work. We plan to sample extensively in other states in the future.

**2. The use of "landraces" as against "cultivars" needs to be justified.**

Landraces are defined as locally adapted, genetically diverse populations of traditional domesticated crops, which are intermediate to cultivated high yielding lines (green revolution lines or cultivars) and wild relatives of crops. Cultivars represent cases where breeders have selectively improved for particular characteristics such as yield. Hence, our rice varieties are better classified as “landraces”. We have now added this explanation to the main text (page 5 lines 103-104).

**3. Line no. 301-306: "We note two caveats here: (1) The 16S rRNA sequence does not provide sufficient resolution to identify.......". Can this be solved by increasing the population size (refer to query 1)? If not how?**

This issue cannot be resolved by increasing population size, because *Methylobacterium* species have very similar 16S rRNA sequences. Thus, sampling more isolates will not be helpful. The only way to resolve strain identity is to obtain more sequence data, e.g. from a larger number of genes or via whole genome sequencing.

**4. Why only Carbon use profile was selected as a parameter for bacterial colonization?**

As the reviewer correctly suggests, other nutrients may also determine bacterial colonization (as indeed, our results suggest). However, carbon availability is a key limiting factor for bacterial metabolism; plant-associated bacteria have more genes involved in carbohydrate metabolism (Levy et al 2017, Nature Genetics); and plant epiphytes typically rely on plant-secreted compounds. Hence we chose to first determine carbon use profiles of rice-associated strains, as the simplest potential determinant of host-bacterial association. We have now included this explanation in the manuscript (page 12, lines 293-298).

**5. Landraces were found to be a good choice for *Methylobacterium* colonization however strain specificity could not be established. And considering the case of Chakhao (Manipur) where temporal variation was least observed (for two-year study), does aromatic character of the cultivar had a role to play?**

It is possible that the aromatic character of Chakhao is important, and we will follow up on this interested idea. However, at the moment we do not have any evidence to support (or refute) this hypothesis.

**6. Does *Methylobacterium* colonization have a negative impact on rice physiology or host deficit or plant defence?**

Thus far, our experiments do not indicate a negative impact on rice hosts.

**Reviewer #2:**

**Appreciate the author and team for working on such important topic but the current manuscript need revision in terms of scientific language.**

It is not clear what aspect of our scientific language requires revision.

**Other changes based on journal requirements**

1. **We note that you have stated that you will provide repository information for your data at acceptance. Should your manuscript be accepted for publication, we will hold it until you provide the relevant accession numbers or DOIs necessary to access your data. If you wish to make changes to your Data Availability statement, please describe these changes in your cover letter and we will update your Data Availability statement to reflect the information you provide.**

We have now uploaded raw data on Figshare, and included the url in the Data Accessibility statement in the manuscript file (page 27). We are uploading 16S rRNA sequence data in Genbank and will make the DOI available on acceptance of the manuscript.

**2. We note that you have included the phrase “data not shown” in your manuscript. Unfortunately, this does not meet our data sharing requirements. PLOS does not permit references to inaccessible data. We require that authors provide all relevant data within the paper, Supporting Information files, or in an acceptable, public repository. Please add a citation to support this phrase or upload the data that corresponds with these findings to a stable repository (such as Figshare or Dryad) and provide and URLs, DOIs, or accession numbers that may be used to access these data. Or, if the data are not a core part of the research being presented in your study, we ask that you remove the phrase that refers to these data.**

We have removed the phrase from the manuscript.
